# Angiopoietin II in Critically Ill Septic Patients: A Post Hoc Analysis of the DRAK Study

**DOI:** 10.3390/biomedicines12112436

**Published:** 2024-10-23

**Authors:** Veronika Bucher, Helen Graf, Johannes Zander, Uwe Liebchen, Danilo Hackner, Caroline Gräfe, Martin Bender, Michael Zoller, Christina Scharf

**Affiliations:** 1 Department of Anaesthesiology, University Hospital, LMU Munich, Marchioninistrasse 15, 81377 Munich, Germany; 2 Labor Dr. Brunner, 78464 Konstanz, Germany

**Keywords:** angiopoietin-2, sepsis, kidney replacement therapy (KRT), extracorporeal membrane oxygenation (ECMO), fluid balance, creatinine clearance

## Abstract

Introduction: Angiopoietin II (Ang-II) plays a pivotal role in the development of microcirculatory dysfunction as it provokes endothelial barrier disruption in patients with sepsis or septic shock. In particular, those with acute kidney injury show high Ang-II concentrations. So far, it is unclear which covariates influence Ang-II concentration in the early phase of sepsis, especially if extracorporeal therapies also do. Methods: Ang-II concentrations were measured in 171 patients with sepsis after the first day of antibiotic treatment between 03/2013 and 01/2015. Ang-II was correlated with potential influencing factors (Spearman correlation). A multivariate model was established including the significant correlating parameters. The Mann–Whitney U test and the Kruskal–Wallis test were used to detect significant differences in Ang-II concentration. Results: The median Ang-II concentration was 8015 pg/mL (interquartile range (IQR): 5024–14,185). A total of forty patients were treated with kidney replacement therapy (KRT) and 20 were supported by venovenous extracorporeal membrane oxygenation (vv-ECMO). Sequential organ failure assessment (SOFA) score (r = 0.541), creatinine clearance (r = −0.467), urinary output (r = −0.289), interleukin (IL)-6 (r = 0.529), C-reactive protein (CRP) (r = 0.241), platelet count (r = −0.419), bilirubin (r = 0.565), lactate (r = 0.322), KRT (r = 0.451), and fluid balance (r = 0.373) significantly correlated with Ang-II concentration and were included in the multivariate model. There, creatinine clearance (*p* < 0.01, b = −26.3, 95% confidence interval (CI) −41.8–−10.8), fluid balance (*p* = 0.002, b = 0.92, 95% CI 0.33–1.51), and CRP (*p* = 0.004, b = 127.6, 95% CI 41.6–213.7) were associated with Ang-II concentration. Furthermore, patients with KRT (median: 15,219 pg/mL, IQR: 10,548–20,270) had significantly (*p* < 0.01) higher Ang-II concentrations than those with vv-ECMO support (median: 6412 pg/mL, IQR: 5246–10,257) or those without extracorporeal therapy (median: 7156 pg/mL, IQR: 4409–12,741). Conclusion: Increased CRP, positive fluid balance, and impaired kidney function were associated with higher Ang-II concentrations in critically ill patients in the early stage of sepsis in this post hoc analysis. In particular, patients with KRT had very high Ang-II concentrations, whereas the use of vv-ECMO was not related to higher Ang-II concentrations. The significance for clinical practice should be clarified by a prospective study with standardized measurements.

## 1. Introduction

Sepsis and septic shock are life-threatening diseases associated with a high mortality of up to 56% [1]. An exaggerated immune response leads to pathogen-mediated release of various cytokines, resulting in damage to the endothelium [2]. This subsequently induces microcirculatory dysfunction and capillary leakage, with the development of circulatory insufficiency and the necessity of vasoactive therapy [3]. A pathologic vascular barrier can be observed in most septic patients; thus, the mechanisms promoting it are still the subject of many studies [4,5,6]. Impairment of the vascular structure and functioning leads to a fluid shift from the vascular space to the interstitial compartment, consequently resulting in edema and impaired pulmonary function [7].

The Angiopoietin-Tie2-Ligand-receptor system plays a pivotal role in the above-described process [8,9,10]. Angiopoietins belong to a family of growth factors responsible for physiological angiogenesis. Their activities are mediated through tyrosine kinase receptors Tie1 and Tie2 [11]. Angiopoietin-I and angiopoietin-II (Ang-I and Ang-II) have a key and opposing function in the entire system [12]. Ang-I mediates the adhesion, migration, and survival of endothelial cells and is critical for vessel maturation. Ang-II, on the other hand, promotes cell death, disconnects the endothelium and perivascular cells, and causes vascular regression [13].

Different authors were able to show that Ang-II alone provokes endothelial barrier disruption, and Ang-II concentrations are elevated in patients with sepsis or septic shock [14,15]. In this context, there might also be an association between Ang-II concentration and patient mortality [16]. However, high Ang-II concentrations are not only observed in patients with sepsis or septic shock. Van der Heijden et al. reported high Ang-II concentrations in ventilated patients with and without sepsis. Circulating Ang-II was associated with patients’ pulmonary permeability, leading to edema [17]. Also, it has been described as an outcome predictor in patients with acute respiratory distress syndrome (ARDS) [18]. One experimental approach might be the blockade of Ang-II to improve survival, which was successfully performed in a murine model [19].

In addition, acute kidney injury (AKI) is associated with high Ang-II concentrations [20]. Kümpers et al. measured the Ang-II concentration in 117 patients shortly before the initiation of kidney replacement therapy (KRT). Ang-II levels were significantly higher in patients with “kidney injury” according to the AKI classification of “RIFLE criteria” (risk (R), injury (I), and failure (F), sustained loss (L) and end-stage kidney disease (E)) and non-survivors [21]. AKI is common in patients with sepsis and up to 37% of these patients require KRT, resulting in a hospital mortality rate of 50–60% [22,23]. A positive correlation between Ang-II concentration and the need for KRT has already been described [24].

This post hoc analysis of a prospective study aims to identify parameters correlating with Ang-II concentrations in the early phase of sepsis and septic shock in intensive care unit (ICU) patients in a multivariate model. In addition, a subgroup analysis was performed based on the results of the multivariate model to investigate whether extracorporeal therapies affect Ang-II concentration.

## 2. Methods

### 2.1. Study Setting

This was a post hoc analysis of a monocentric, prospective, and observational study investigating the correlation between Ang-II and clinical and laboratory parameters in ICU patients with sepsis or septic shock. Patients treated at two anesthesiologic ICUs at the Ludwig Maximilians University (LMU) hospital in Munich were included between 1 March 2023 and 31 January 2015. The local institutional review board approved this study (registration number: 428-12 on 13 November 2012). This study was registered at clinicaltrials.gov (NCT 01793012). Furthermore, written informed consent was obtained from the patients or their legal representatives before their inclusion in this study. The inclusion criteria were an age of ≥18 years and diagnosed sepsis or septic shock with an antibiotic treatment.

### 2.2. Laboratory Measurements and Data Collection

All clinical and chemical parameters were determined using standard clinical chemistry tests at the Institute of Laboratory Medicine, LMU, Munich. Serum samples for the determination of Ang-II were collected from the arterial line one day after starting antibiotic therapy due to sepsis or septic shock. The samples were immediately sent to the laboratory, centrifuged (3000× *g*, 10 min), and aliquoted into 2 mL polypropylene tubes (Eppendorf, Hamburg, Germany). Serum aliquots were stored within one hour after blood sampling at −80 °C. Ang-II was measured with an Enzyme-Linked Immunosorbent Assay (ELISA) (Quantikine ELISA Human Ang-II Immunoassay, R&D Systems, Minneapolis, MN, USA) and quantified with Spectramax Paradigm of Molekular Devices (San Jose, CA, USA).

Demographic data as well as clinical and laboratory variables were collected from the laboratory and patient information system (SAP Krankenhausinformationssystem i.s.h. med^®^ and QCare PDMS, HIM^®^, version 2012, Bad Homburg, Germany). The baseline data (age, weight, gender, sequential organ failure assessment (SOFA) score, KRT, venovenous extracorporeal membrane oxygenation (vv-ECMO), ARDS, urinary output, fluid balance, and source of infection) were collected upon inclusion in this study. All laboratory data (i.e., creatinine, albumin, C-reactive protein (CRP), interleukin-6 (IL-6), leukocytes, thrombocytes, bilirubin, cholinesterase, and lactate) were collected the day after starting antibiotic treatment.

### 2.3. Statistical Analysis

Statistical analysis was performed using IBM SPSS Statistics (IBM SPSS Statistics for Windows, Version 26.0, IBM Corp, released 2023, Armonk, NY, USA). The median with the interquartile range (IQR) was calculated. Ang-II concentration was correlated with different parameters (age, sex, body mass index (BMI), SOFA score, creatinine clearance, dialysis, vv-ECMO, urinary output, fluid balance, albumin, IL-6, CRP, ARDS, leukocytes, thrombocytes, bilirubin, cholinesterase, lactate, and 28-day mortality) by using the Spearman correlation coefficient. A *p*-value of *p* < 0.0026 (Bonferroni correction for multiple testing, alpha level of 0.05) was considered statistically significant. A generalized linear model (GLM) was employed by including the parameters that correlated significantly with Ang-II concentration. The Mann–Whitney U test or Kruskal–Wallis test were used to compare different subgroups.

## 3. Results

### 3.1. Demographic and Clinical Data

A total of 171 patients were included in this study. The median age was 58 years and 63% were female. The source of infection leading to sepsis or septic shock in descending order was as follows: pneumonia (*n* = 121, 70.8%), abdominal infection (*n* = 35, 20.5%), catheter line-associated infections (*n* = 5, 2.9%), endocarditis or urinary tract infection (*n* = 4 each, 2.3% each), and bone infection (*n* = 2, 1.2%). Overall, 40 patients needed KRT, and 20 patients received vv-ECMO support. A total of 41 patients (24.0%) suffered from ARDS and 60 patients were treated after solid organ transplantation (lung: 43 (25.1%); liver: 17 (9.9%)). Detailed patient characteristic and laboratory parameters are displayed in Table 1.

### 3.2. Correlation Analysis and GLM

Spearman correlation analysis was performed for Ang-II and different parameters. A significant (*p* < 0.0026) correlation was observed between Ang-II and SOFA score (r = 0.541), creatinine clearance (r = −0.467), urinary output (r = −0.289), IL-6 (r = 0.529), CRP (r = 0.241), thrombocytes (r = −0.419), bilirubin (r = 0.565), lactate (r = 0.322), KRT (r = 0.451), and fluid balance (r = 0.373). All other parameters showed no significant correlation with Ang-II concentration. Supplementary Figures S1–S9 illustrate the Ang-II concentration and the significantly correlating parameters.

A GLM was employed with Ang-II as the dependent variable and the above-mentioned significant correlating parameters as potential covariates. In the multivariate model, three parameters were identified as significant determinants of Ang-II concentration. Lower creatinine clearance (in patients without KRT) is related to a significantly higher Ang-II concentration (*p* < 0.01, b = −26.3, 95% CI −41.8–−10.8). In contrast, a more positive fluid balance was associated with significantly higher Ang-II concentrations (*p* = 0.002, b = 0.92, 95% CI 0.33–1.51). Last, a higher CRP concentration was also associated with higher Ang-II concentrations (*p* = 0.004, b = 127.6, 95% CI 41.6–213.7).

### 3.3. Influence of Fluid Balance, CRP, and Kidney Function on Ang-II Concentration

**Figure 1:**
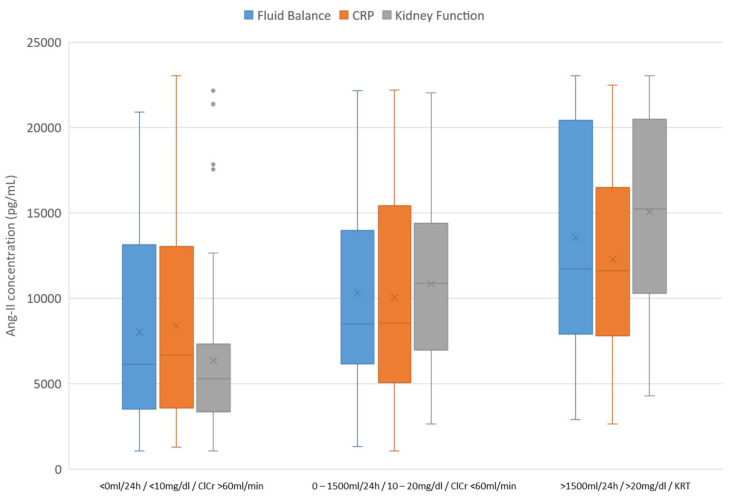
Ang-II concentrations in three different subgroups (fluid balance, CRP, kidney function). Note: The first three boxplots include patients with a fluid balance < 0 mL/24 h (blue), CRP < 10 mg/dL (orange), and creatinine clearance > 60 mL/min (gray). The second three boxplots include patients with a fluid balance 0–1500 mL/min (blue), CRP 10–20 mg/dL, and creatinine clearance < 60 mL/min (gray). The last three boxplots include patients with a fluid balance > 1500 mL/24 h, CRP > 20 mg/dL (orange), and kidney replacement therapy (gray). The boxes of the boxplots represent the interquartile range (IQR) and the horizontal line represents the median. The whiskers are limited to 1.5 times the IQR. The mean is indicated by the cross.

Patients were divided into three groups based on their fluid balance in the last 24 h before Ang-II measurement (<0 mL/24 h, 0–1500 mL/24 h, >1500 mL/24 h), CRP concentration (<10 mg/dL, 10–20 mg/dL, >20 mg/dL), and kidney function (creatinine clearance > 60 mL/min, creatinine clearance < 60 mL/min, KRT). The Kruskal–Wallis test showed an overall significant (*p* < 0.01) difference in Ang-II concentration in the three different subgroups. Table 2 shows the Ang-II concentrations in the different subgroups. Figure 1 illustrates the Ang-II concentrations in the different subgroups as boxplots.

### 3.4. Influence of Extracorporeal Therapy on Ang-II Concentrations

**Figure 2:**
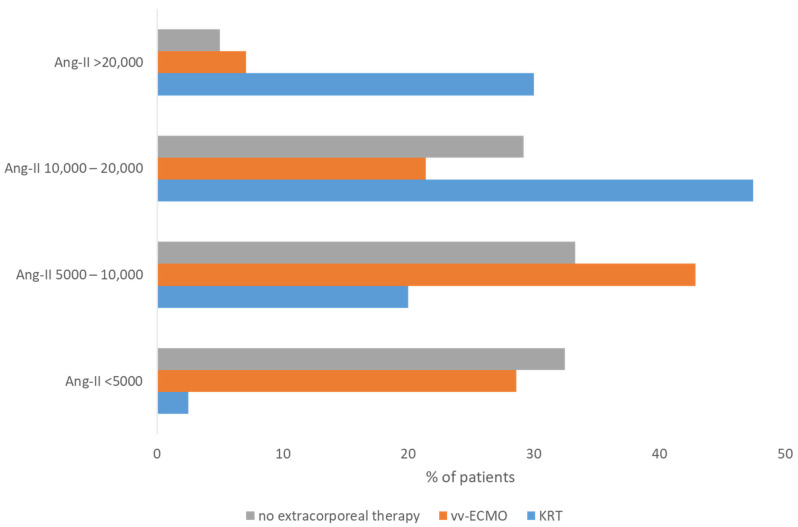
The percentage of patients with Ang-II concentrations < 5000, 5000–10,000, 10,000–20,000, and >20,000 pg/mL in the subgroups with KRT, vv-ECMO, and no extracorporeal therapy.

A total of 40 patients were treated with KRT and 14 patients received vv-ECMO support without KRT. There was an overall significant (*p* < 0.01) difference between the three groups: KRT, vv-ECMO without KRT, and no extracorporeal therapy. Patients with KRT had significantly (*p* < 0.01) higher Ang-II concentrations (median: 15,219 pg/mL; IQR: 10,548–20,270) compared to those with vv-ECMO therapy (median: 6412 pg/mL; IQR: 5246–10,257) and those with no extracorporeal therapy (median: 7156 pg/mL; IQR: 4409–12,741). No significant difference was observed between vv-ECMO therapy and no extracorporeal therapy. Figure 2 illustrates the Ang-II concentrations in the above-mentioned groups.

## 4. Discussion

The management of patients with sepsis and septic shock remains challenging in intensive care medicine. New biomarkers that help to improve patients’ outcomes would be desirable. Various authors have already described the correlation between sepsis and high Ang-II concentrations [25,26]. We were able to demonstrate a positive correlation between Ang-II concentration and CRP in our multivariate model. The higher the CRP concentration and therefore the more severe the infection, the higher the Ang-II concentrations were. Contrary to what previous authors have described [9,15], there was no association with 28-day mortality or SOFA score as a measure for disease severity. In principle, one explanation for this could be that the initial inflammatory response has no bearing on the outcome, as this depends on other parameters such as the choice of the right anti-infective agent and the patient’s previous illness.

Moreover, our data show a positive correlation between fluid balance and Ang-II concentration. As high Ang-II concentrations indicate endothelial disintegration [27], with the recruitment of small capillaries [28], a positive fluid balance might reflect fluid leakage into the interstitium. Volume therapy is a necessary therapeutic option for treating the associated circulatory insufficiency, which promotes the development of edema and volume overload [29,30]. Ang-II, if measured daily, could serve as a marker for endothelial barrier disruption and thus indirectly provide information on whether a negative balance is possible or not. Routine measurement was recently recommended by Atreya at al. to strengthen its role as a predictor and therapeutic approach in the future [31].

We observed a significant negative correlation between creatinine clearance and Ang-II concentration. This is not new information and has already been described by various authors [32,33]. However, our data indicate that Ang-II concentrations are significantly higher in patients with KRT compared to patients with impaired kidney function without KRT. While renal failure requiring dialysis often occurs in association with sepsis, Robinson-Cohen et al. highlighted that Ang-II elevation is independent of inflammation in the context of renal failure [20]. Furthermore, recent data highlight the role of Ang-II as the best biomarker for the prediction of AKI in patients with sepsis and respiratory failure [33].

Different authors describe an association between pulmonary insufficiency and high Ang-II concentrations [17,34,35]. Our study included 14 patients with vv-ECMO and associated severe ARDS. We observed no difference between the Ang-II concentrations in patients with vv-ECMO and those with no extracorporeal therapy. As this subgroup includes fewer patients, this information must be interpreted with caution and should be further investigated. However, blocking Ang-II in patients with pneumonia was not associated with less necessity for ventilation in a randomized trial [36]. It must be critically noted that Ang-II was not measured and the patients were not critically ill suffering from sepsis. As less destruction of lung epithelial cells was observed in rats with LPS-induced lung injury and an Ang-II blockade [37], further research should focus on critically ill patients with microcirculatory dysfunction, including the measurement of Ang-II.

In conclusion, the presented data offer a foundation for further investigations to explore the possible role of Ang-II as a biomarker in routine clinical practice and therapeutic options of blocking Ang-II. Ang-II levels should be investigated in patients with septic and non-septic acute renal failure during the course of treatment to further evaluate the effect of dialysis on Ang-II concentration. As recent data highlight its role in the development of acute or chronic liver failure, patients with liver dysfunction with and without prior liver impairment should be examined [38]. One should study whether the course of Ang-II concentration can predict the possibility of a negative balance in patients with capillary leakage. Its concentration should be further evaluated if non-septic ARDS in particular is associated with an increase in Ang-II concentrations, and the diagnostic value of Ang-II and its causative role in patients with ARDS should be determined [39]. As hyperglycemia is associated with higher Ang-II expression in vitro, this potential influencing factor should also be investigated in critically ill patients [40].

This study has some relevant limitations. First, only one Ang-II concentration was measured after the initiation of antibiotic treatment in patients with sepsis or septic shock. Thus, no statement can be made about the progression of Ang-II during therapy. Second, the measurement did not take place before the start of antibiotic therapy, but on the day after initiation. This non-standardized timing has to be mentioned as another limitation. Furthermore, no statement can be made about the concentration at the start of antibiotic therapy. Since Ang-I was not measured, the Ang-I/Ang-II ratio cannot be determined in our study. However, Ang-II alone seems to be a valid parameter for assessing endothelial function [33]. Last, correlation analysis is not the most robust statistic method; however, a multivariate model has the potential to identify actual influencing factors. As some subgroups (i.e., patients with vv-ECMO) were quite small, it precludes drawing firm conclusions about these important subgroups. Therefore, focusing on those in a prospective study seems reasonable.

## 5. Conclusions

In our post hoc analysis, higher Ang-II concentrations were detected in ICU patients with higher CRP levels, a positive fluid balance, and impaired kidney function in the initial phase of sepsis. In particular, patients with KRT had very high Ang-II concentrations. In contrast, patients with vv-ECMO did not have higher Ang-II concentrations compared to patients without extracorporeal therapy. The role of Ang-II as a potential biomarker for ICU patients with sepsis and acute renal failure should be evaluated in a prospective study with standardized measurements.

## Figures and Tables

**Table 1:** Patient characteristics and laboratory measurements.

	*n* (%) or Median [IQR]
Patient characteristics	
Age (years)	58 [48, 67]
Gender: male/female	64 (37.4)/107 (62.6)
BMI (kg/m^2^)	24.8 [21.9, 29.1]
28-days mortality	24 (14.0)
SOFA Score	12 [9, 15]
KRT	40 (23.4)
vv-ECMO support	20 (11.7)
Urinary output (mL/24 h)	2600 [375, 3975]
Fluid balance (mL/24 h)	325 [−610, 1560]
Laboratory parameters	
Albumin (g/dL)	2.7 [2.4, 3.0]
Interleukin-6 (pg/mL)	99 [49, 304]
C-reactive protein (mg/dL)	13.4 [7.8, 20.3]
Creatinine-Clearance (mL/min) in patients without KRT	73 [34, 108]
Leucocytes (G/L)	13.8 [8.7, 20.2]
Thrombocytes (G/L)	155 [103, 245]
Bilirubin (mg/dL)	1.0 [0.6, 2.9]
Cholinesterase (kU/L)	3.1 [2.1, 4.3]
Lactate (mmol/L)	1.5 [1.1, 2.5]
Angiopoietin-II (pg/mL)	8015 [5024, 14,185]

Note: BMI: body mass index; SOFA: sequence organ failure assessment; KRT: kidney replacement therapy; vv-ECMO: venovenous extracorporeal membrane oxygenation. The angiopoietin-II reference area in healthy controls: 2000–3000 pg/mL. All parameters were collected the day after starting antibiotic therapy due to sepsis or septic shock.

**Table 2:** Angiopoietin II concentration (pg/mL) in different subgroups.

	Median (IQR)
Fluid Balance	
<0 mL/24 h	6124 (3559–13,029)
0–1500 mL/24 h	8472 (6269–13,900)
>1500 mL/24 h	11,718 (7915–20,237)
CRP	
<10 mg/dL	6656 (3591–12,398)
10–20 mg/dL	8537 (5065–15,140)
>20 mg/dL	11,604 (7806–15,147)
Kidney Function	
ClCr > 60 mL/min	5292 (3371–7207)
ClCr < 60 mL/min	10,856 (7219–14,264)
KRT	15,219 (10,548–20,270)

Note: IQR: interquartile range; CRP: C-reactive protein; ClCr: creatinine clearance (mL/min); KRT: kidney replacement therapy.

## Data Availability

All data generated during this study are included in this article.

## Supplementary Materials

The following supporting information can be downloaded at: https://www.mdpi.com/article/10.3390/biomedicines12112436/s1, Figure S1: Correlation between Ang-II (pg/mL) and SOFA Score; Figure S2: Correlation between Ang-II (pg/mL) and creatinine clearance (mL/min); Figure S3: Correlation between Ang-II (pg/mL) and urinary output; Figure S4: Correlation between Ang-II (pg/mL) and interleukin-6 (pg/mL); Figure S5: Correlation between Ang-II (pg/mL) and CRP (mg/dL); Figure S6: Correlation between Ang-II (pg/mL) and thrombocytes (G/L); Figure S7: Correlation between Ang-II (pg/mL) and bilirubin (mg/dL); Figure S8: Correlation between Ang-II (pg/mL) and lactate (mmol/L); Figure S9: Correlation between Ang-II (pg/mL) and fluid balance (mL/24 h).
