# Supplementary material for: Angiopoietin II in Critically Ill Septic Patients: A Post Hoc Analysis of the DRAK Study
Source: Biomedicines. 2024 Oct 23;12(11):2436. doi: 10.3390/biomedicines12112436 (PMC11591998; doi:10.3390/biomedicines12112436)

Supplemental File

Figure S1: Correlation between Ang-II (pg/mL) and SOFA Score

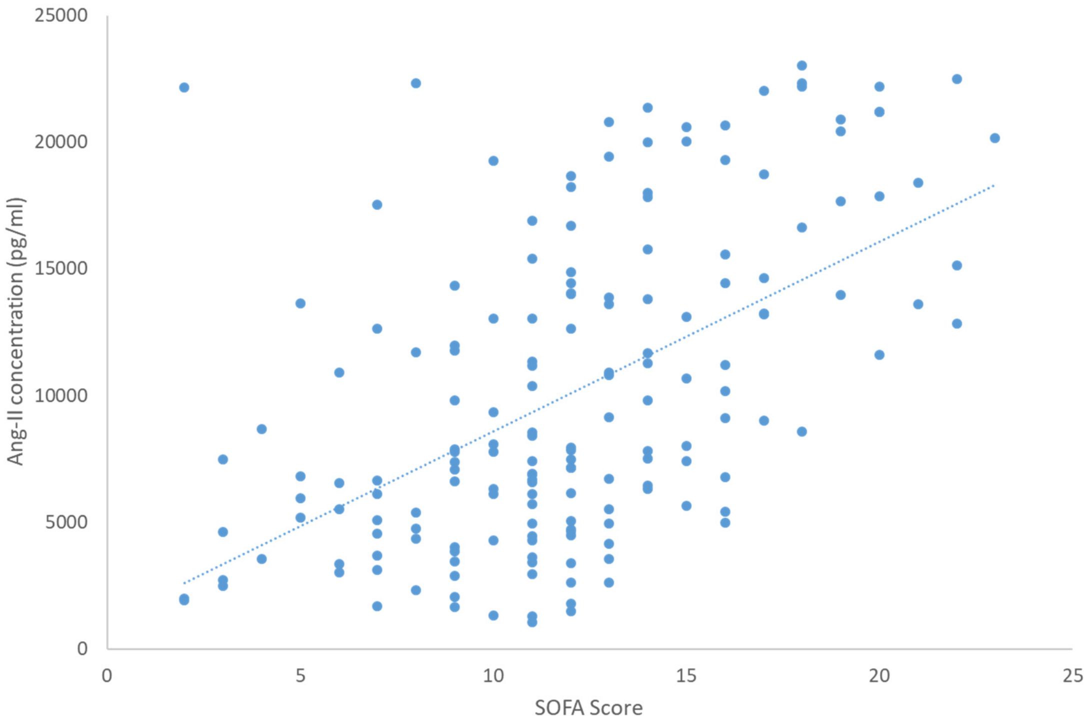

Figure S2: Correlation between Ang-II (pg/mL) and creatinine clearance (mL/min)

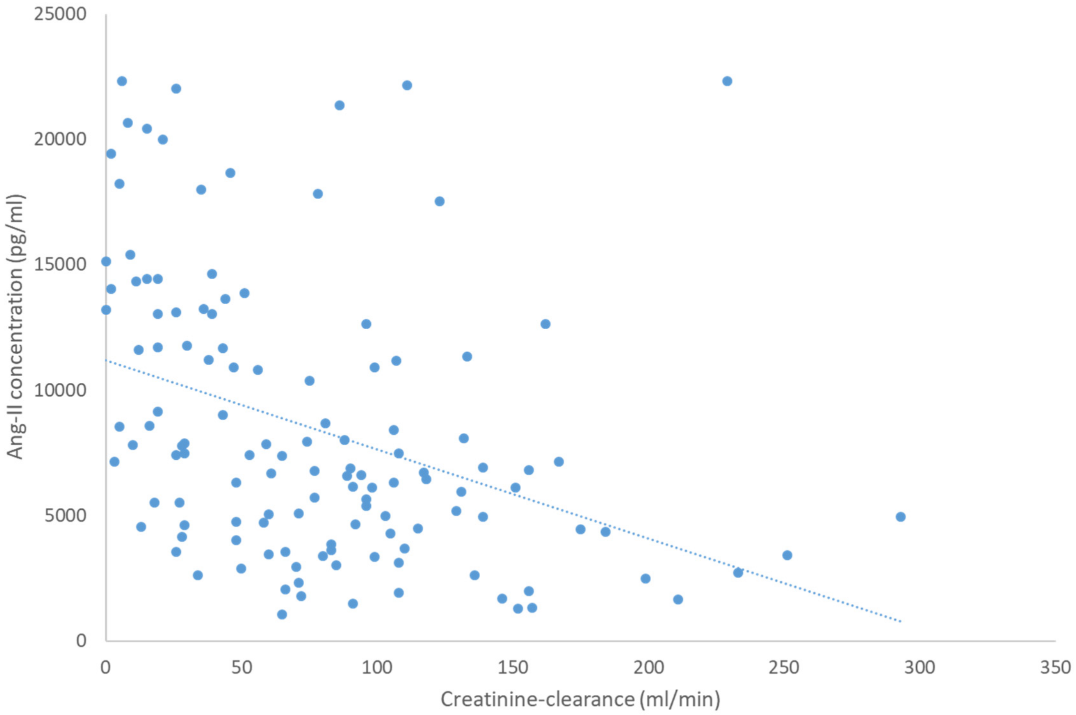

Figure S3: Correlation between Ang-II (pg/mL) and urinary output

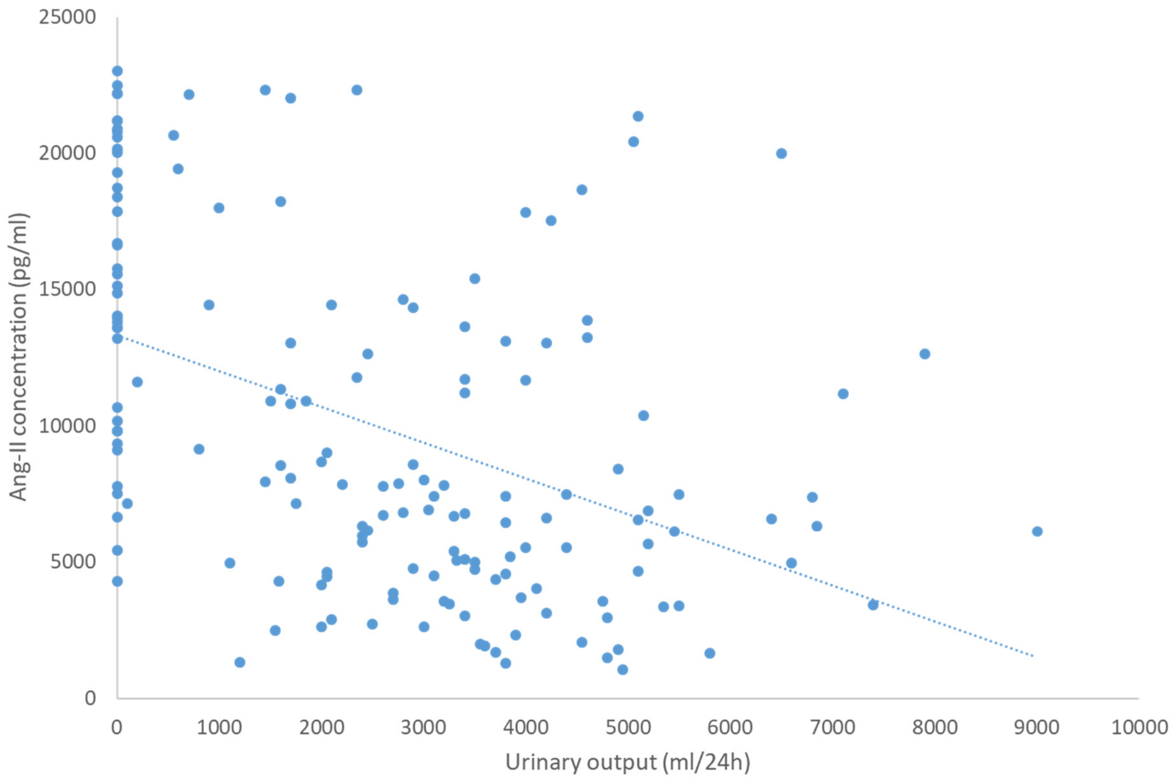

Figure S4: Correlation between Ang-II (pg/mL) and interleukin-6 (pg/mL)

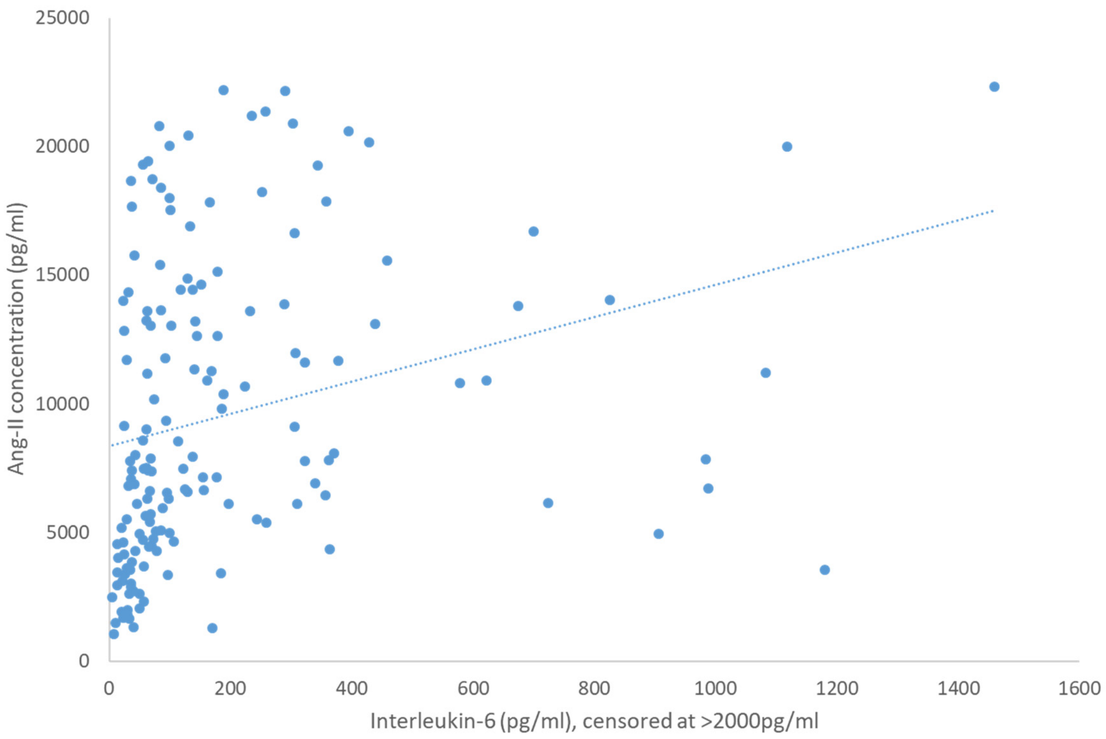

Figure S5: Correlation between Ang-II (pg/mL) and CRP (mg/dL)

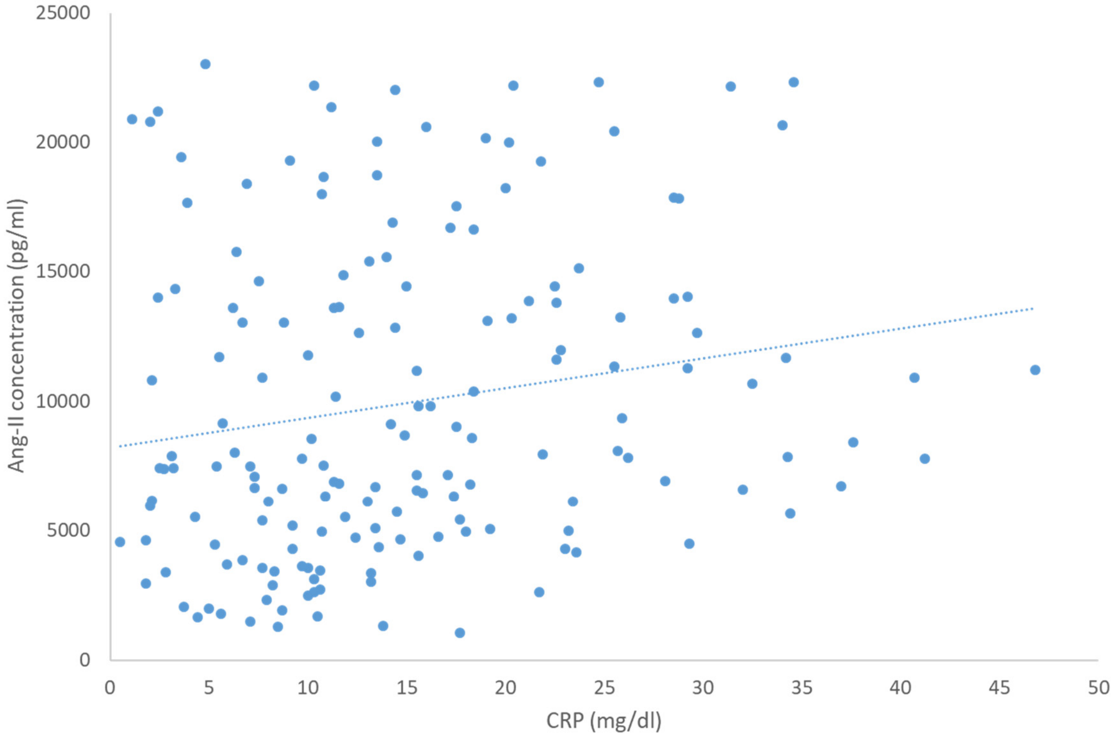

Figure S6: Correlation between Ang-II (pg/mL) and thrombocytes (G/L)

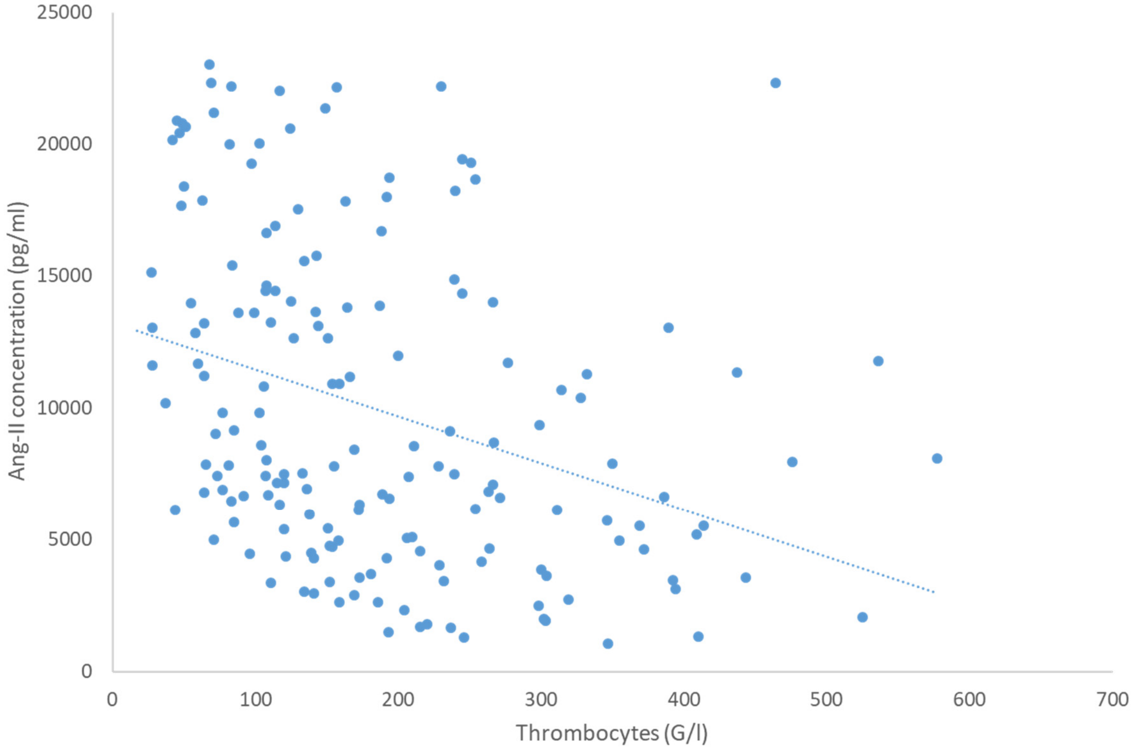

Figure S7: Correlation between Ang-II (pg/mL) and bilirubin (mg/dL)

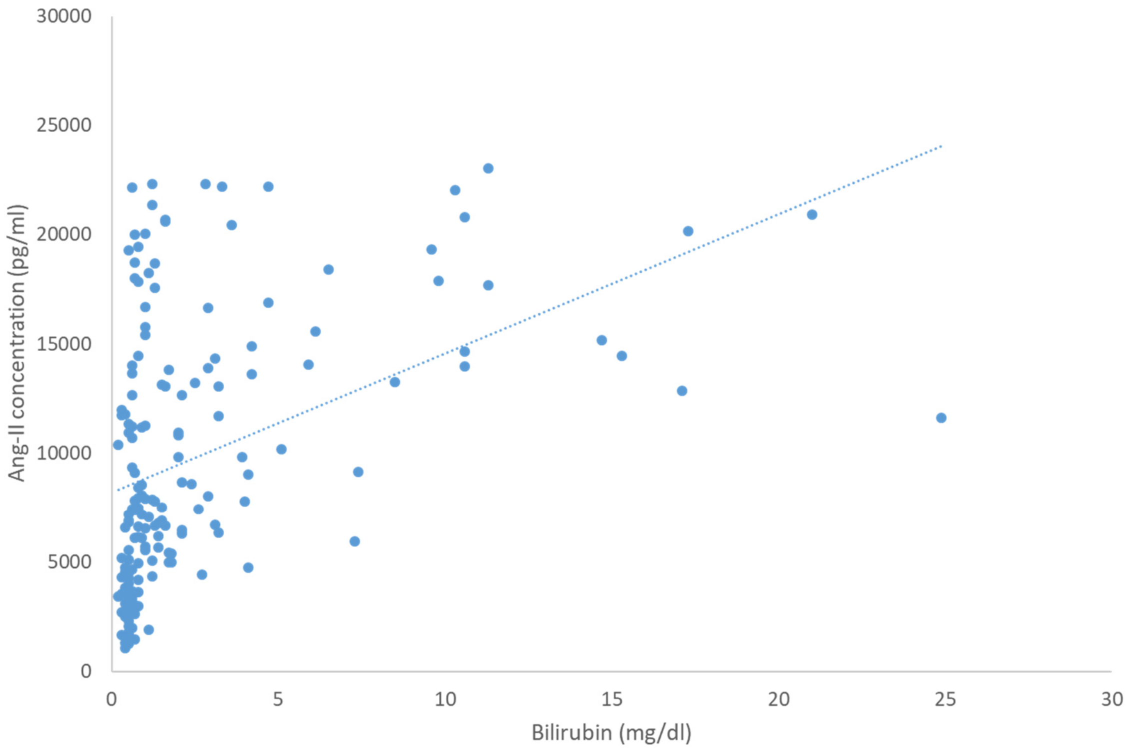

Figure S8: Correlation between Ang-II (pg/mL) and lactate (mmol/L)

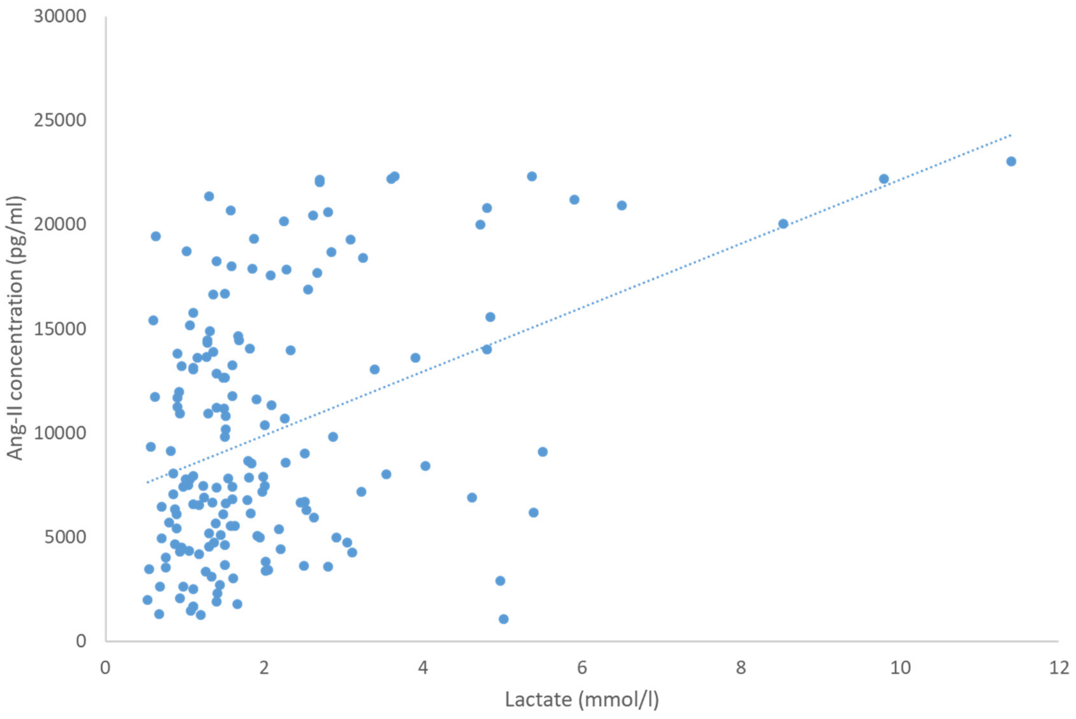

Figure S9: Correlation between Ang-II (pg/mL) and fluid balance (mL/24h)

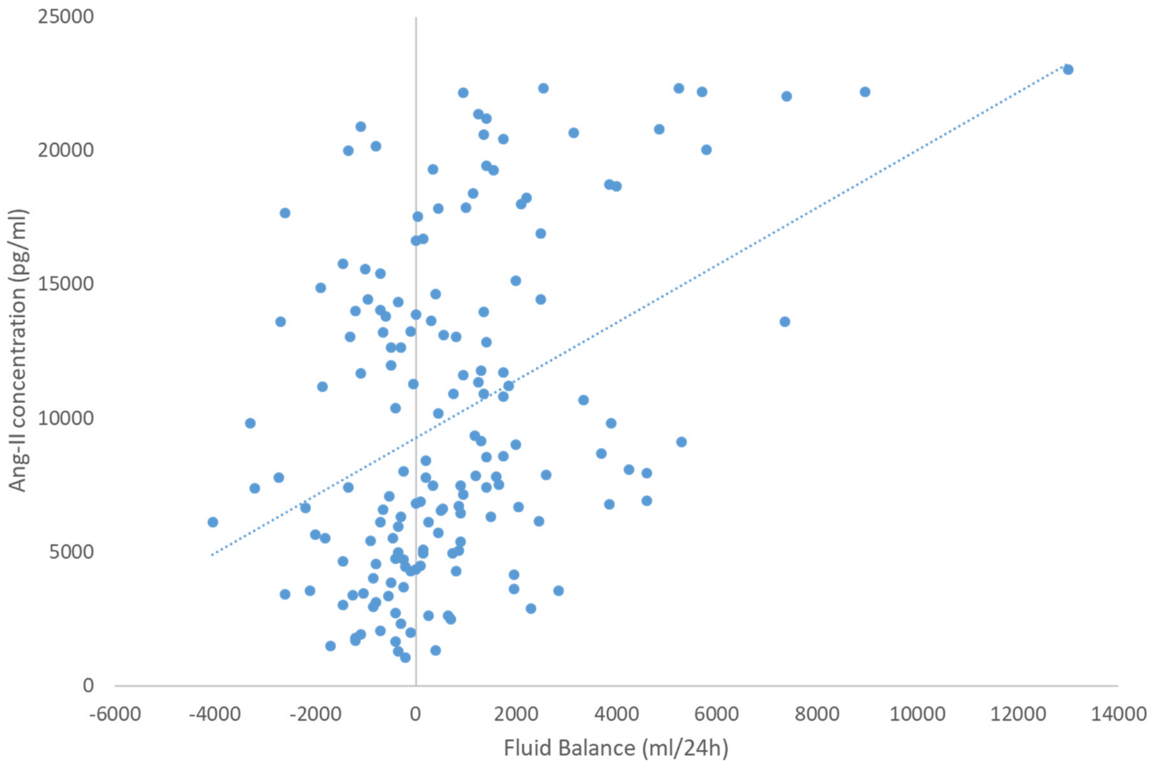

Supplement: Supplementary file 1 [file biomedicines-12-02436-s001.zip › biomedicines-3238612-supplementary.pdf]
